# An exploratory study on the possibilities of microalgal biotechnology to obtain the essential ^6^Li isotope as fusion fuel

**DOI:** 10.1186/s13068-023-02394-0

**Published:** 2023-09-21

**Authors:** Camino García-Balboa, Paloma Martínez-Alesón, Victoria López-Rodas, Eduardo Costas Costas, Marta Fernández Díaz

**Affiliations:** 1 School of Veterinary Medicine, Complutense University of Madrid, Av. Puerta de Hierro s/n, 28040 Madrid, Spain; 2 Spanish Research Centre for Energy, Environment and Technology (CIEMAT), Av. Complutense 40, 28040 Madrid, Spain

**Keywords:** Fusion energy, Microalgae, Lithium, Enrichment, Metal uptake

## Abstract

Future energy supply needs to overcome two challenges: environmental impact and dependence on geopolitically unstable countries. A very promising alternative is based on lithium, an element for batteries, and whose isotope ^6^Li will be essential in nuclear fusion. The objective of this research has been to determine if it is possible to achieve isotopic fractionation of lithium through a process mediated by microalgae. For this purpose, *Chlamydomonas reinhardtii* was selected and grown in presence of 5 mg/L of lithium. Results revealed that this specie survives at the selected lithium concentration, discriminates isotopes and preferentially capture ^6^Li (^6^δ = 10.029 ± 3.307) through a process independent of the cellular growth. Concomitate recovered up 0.206 mg/L of lithium along a process of 21 days. The result of this study lets to affirm that *Chlamydomonas reinhardtii* might be used to obtain lithium enriched in the lighter isotope.

## Introduction

The human population is expected to reach around 10 billion of individuals in the next 2050. The energy requirements of this exponentially growing population and high technology consumers must be consistent with this expectation if the actual lifestyle is maintained. The consequences of global change, well defined by OMS and discussed in the COP27 (Egypt 2022), make necessary look forward to new energetic sources more preservative with the health of the planet. In addition, it will be necessary to get a stable supply of energy sources to avoid the geopolitical uncertainty associated with the main provider countries.

Setting our sights on these aspects, one alternative for future energy development is nuclear fusion power. The conceptual basis of this process is the fusion of the two heaviest isotopes of hydrogen, deuterium (D) and tritium (T), through a reaction in which energy and one proton are released Eq. (1).

1 $${}_{1}{}^{2}\mathrm{H}(\mathrm{D})+{}_{1}{}^{3}\mathrm{H}(\mathrm{T})\to 4\mathrm{He}+\mathrm{n}+17.6\mathrm{MeV}$$

2 $${}_{3}{}^{6}\mathrm{Li}+{}_{0}{}^{1}\mathrm{n}\to {}_{1}{}^{3}\mathrm{H}(\mathrm{T})+{}_{2}{}^{4}\mathrm{He}$$

The emitted neutron is the carrier of much of the energy of the reaction in the form of kinetic energy, which can be used by transferring it to the reactor envelope in the form of heat. This envelope is covered with ^6^Li so when the released neutron hits the surface, in addition to the transfer of kinetic energy, the reaction according to Eq. (2) takes place, resulting tritium is regenerated.

Although this technology is yet in progress, an international research consortium (International Thermonuclear Experimental Reactor, ITER) was stablished in 2007 with the objective to design operating conditions for the fusion reaction.

As seen in Eq. (1), the fuels need to keep the fusion are D and T. D is a relatively frequent isotope, with a seawater concentration of 33 g/m^3^ [1] whereas T is a scarce isotope with only around 20 kg of reserves in the world [2]. The supply of T may come from ^6^Li Eq. (2), the stable and lighter isotope of Li, found naturally on Earth with a relative frequency of 7.59% [3]. To carry out the sustained fusion reaction, enrichments on this isotope of the order of tens of % are required. Specifically, the enrichment required to get a "fusion grade lithium” varies from 30 to 60% for solid and up to 90% for liquid regenerative coatings, respectively [4, 5].

The relative urgency to obtain isotopes of lithium has led to search for alternative developments for the isotopic enrichment of this element in recent years. All the approaches are based on physicochemical fundamentals at laboratory scale such as electrochemical separation studies [6, 7], laser [8], extraction with ethers or ionic liquid systems [9, 10], chromatography [11] and membrane separation [11, 12].The only strategy implemented at industrial scale is the named Colex process, an environmentally dangerous method that requires large amounts of mercury where ^6^Li accumulates. At the present, the lithium enrichment is almost restricted to China and it is done through this process [13].

All these reasons justify the relevance of looking for other alternatives to obtain ^6^Li. Isotope separation is a process that requires specificity because it involves the challenge of separating two atoms that only differ in their mass number. The ability to separate isotopes (isotope discrimination) is not a phenomenon foreign to living beings. In fact, this possibility has been described in prokaryotes, eukaryotes and even mammalian cells. The microbial fractionation of elements is described in elements as N and O [14]; Hg [15]; N and C [16, 17]; sulphur [18] or copper [19]. Regarding lithium, there is only a descriptive research on the possibilities of several bacterial groups to discriminate between lithium isotopes [20].

Although many biotechnological applications of fungi, bacteria and microalgae have been described for a wide variety of purposes, ranging from biopiles [21] to the remediation of sites contaminated with metals [22] or bioleaching [23], almost nothing has been explored looking for the isotopic fractionation with energy purposes. Only, it has been proved the capacity of microalgae to discriminate between ^235^ and ^238^U [24].

With the focus on energy-related applications, to determine the possibilities of microalgal lithium discrimination, it was selected the microalga *Chlamydomonas reinhardtii*, a model organism with low energy and material requirements. In an exploratory research, early signs of microalgal lithium fractionation were founded [25]. To confirm this result and deepen the kinetic and yield aspects, a strict methodological design was performed to: (i) quantify the discrimination factor (^6^δ) (ii) evaluate their relation with cellular growth and (iii) compare ^6^Li/^7^Li relation with a standard chemical control. These aspects had not been considered before. Complementarily, the capacity of *Chamydomonas reinhardtii* to uptake lithium and its relation with microalgal growth was also evaluated.

## Materials and methods

### Microalgal strain and culture conditions

For the experiments it was used a Chlorophyte obtained from the Microalgae Culture Collection of the research group Albiotox (University Complutense of Madrid, Spain). Specifically, it was selected the specie *Chlamydomonas reinhardtii* Dangerad (Chlamy) isolated from Doñana National Park (Andalucía, Spain). For its maintenance in the laboratory conditions, as described in [26], the strain was transferred axenically every 20 days to maintain the mid-log exponential growth and cultured with 20 mL of bi-distilled water enriched with BG-11 standard broth (Sigma-Aldrich^®^, Chemie,Taufkirchen, Germany) in 50 mL cell culture flasks (Greiner, Bio-OneInc., Longwood, NJ). The cell cultures were grown under continuous light conditions at 80 µmol m^−2^ s^−1^ over the waveband 400–700 nm, with a controlled temperature of 22 °C ± 2 °C.

### Experimental design

The trial was designed to obtain comparative data from: (1) cultures of *Chlamydomonas reinhardtii* in presence of lithium (ChlamyLi); (2) cultures of *Chlamydomonas reinhardtii without* lithium (biological control) and (3) the evolution of Li in the same culture conditions but without microalgal cells (chemical control). Three replicates of each experimental group were established. Cultures with lithium were prepared in 500 mL cell culture flasks (Greiner, Bio-OneInc., Longwood, NJ). Culture volume of 200 mL each one consisted in BG-11 culture media (Sigma-Aldrich^®^, Chemie, Taufkirchen, Germany) supplemented with LiCl (Sigma-Aldrich, BioXtra, 99.0% purity) up to a concentration of 5 mg/L. Each grainer was inoculated with 50,000 cells/mL of *Chlamydomonas reinhardtii*. The biological control replicates were prepared in the same way, but LiCl was not added in this case. The chemical control consisted in BG-11 culture media supplemented with LiCl (5 mg/L) and without microalga. All vials were preserved in a culture camera in continuous light conditions of 80 µmol m^−2^ s^−1^ over the waveband 400–700 nm, and with a controlled temperature of 22 ± 2 °C.

Each vial was sampled (10 mL) to carry out the analysis for monitoring variables. Sampling times considered were the following: 0 (starting moment, just to put in contact cells, culture media and LiCl, when necessary), 7, 14 and 21 days. Three variables were studied for each sampling day: the total number cells; the concentration of lithium in pellets, and the isotope relation ^6^Li/^7^Li in pellets and supernatants (in samples from biological cultures) and in solutions (samples of chemical controls).

### Analysis

#### Counting cells

The total number of cells was estimated with a Beckman Coulter Z2 cell counter (Beckman Coulter^®^ Inc. Particle Characterization Group, Florida). Dilutions for the measurements were prepared in 9.9 mL of ISOTON^®^ commercial liquid with an inoculum of 0.1 mL of each coulter.

### Lithium quantification and isotopic fractionation analysis

Lithium concentration was measured in microalgal pellets and in solutions. Prior to analysis, cultures of microalgal (experimental set and biological control) were centrifuged at 4,000 rpm for 15 min. Cellular pellets and supernatants were frozen at −40 °C and preserved until analysis. Samples from chemical control were also preserved at −40 °C. All chemical determinations were performed at the same time in analogue conditions at CIEMAT (Spanish Research Centre for Energy, Environment and Technology).

### Lithium concentration determination

The measure of lithium concentration was performed following reported details [25]. The lithium concentration was determined by Inductively Coupled Plasma Mass Spectrometry (ICP-MS) using a quadrupole instrument equipped with a collision cell (iCAP Q, Thermo Fisher Scientific Inc., Waltham, MA, USA) and by applying the external calibration quantification method and internal standardization. The standard calibration solutions were prepared daily by dilution from a 1000 mg/L Li stock solution. According to the concentration obtained in each sample, these were diluted to a Li content of 1–2 ng/mL.

For the quantification of Li in pellets, previously to instrumental analysis, algal pellets were subjected to acid digestion with 5 mL of a HNO_3_-H_2_O_2_ mixture (4:1 v/v) in Teflon beakers and gentle heating on a hotplate until a transparent solution was achieved. Sample solutions were then evaporated until almost dry, and the residues were dissolved in 3 M HNO_3_. One milliliter of this solution was brought to a final volume of 10 mL with Milli-Q water to quantify Li by quadrupole-based ICP-MS (Q-ICP-MS). The drying operation was repeated, with the remaining volume and the residue being dissolved in 2 mL of HCl 0.2 M, which was used for lithium isotopic analysis after lithium purification through ion exchange chromatography.

### Quantification of ^6^Li/^7^Li ratio

Isotope compositions were measured on an Element 2 ICP-MS (Thermo Fisher Scientific). Tuning parameters were adjusted before the analyses to maximize instrument sensitivity and stability. The experimental procedure to determine the ^6^Li/^7^Li ratio followed previously reported manuscript [25].

### Chromatography

The separation of lithium from the matrix is crucial for precise Li isotope measure. This step was conducted in a single-step chromatographic separation using 1.5 cm polypropylene columns (Bio-Rad Laboratories, Inc., Hercules, CA, USA) packed with cation-exchange resin, Dowex 50W-X8 (50–100 mesh size).

Prior to sample loading, the columns were pre-washed with 20 mL of 6 M HCl and then conditioned with 10 mL of 0.2 M HCl. Samples in 0.2 M HCl medium were loaded and were subsequently eluted with 40 mL of 0.2 M HCl, collecting the eluted volume ranging from 20 to 32 mL of the corresponding to lithium fraction. These 12 mL were gently evaporated until dry, after which the residue was dissolved in 5 mL of 2% HNO_3_ (v/v). The lithium concentration of these solutions was quantified, and its concentration was adjusted by dilution in 2% HNO_3_ to approximately 1 ppb before isotopic mass spectrometry analysis.

### Mass spectrometry

The ^6^Li/^7^Li ratio was measured using a sample-standard bracketing method, where a blank and a certified isotopic standard (IRMM-016, Joint Research Centre European Commission) were measured before and after each sample to correct for the instrumental drift and mass bias.

The standard isotope was prepared by matching its concentration with that of the sample, that is, 1 µg/L, because an uneven concentration between the sample and the bracketing standards affects the accuracy of the Li isotope analysis. Additionally, blanks were systematically determined and were negligible. The uncertainty associated with the measurement of all ^6^Li/^7^Li isotopic ratios was less than their variation between samples.

The discrimination factor (δ^6^) was estimated according to the equation [3].

3 $${\delta }^{6}(\permil )=\left(\frac{\left(\frac{6Li}{7Li}\right)pellet}{\left(\frac{6Li}{7Li}\right)reference}-1 \right)x 1000$$

To estimate δ^6^ in pellets and supernatants, the value of ^6^Li/^7^Li in supernatants was considered as reference; when compare pellets and chemical control, the ^6^Li/^7^Li from chemical control was the reference.

## Results and discussion

This research was directed to elucidate if *C. reinhardtii* may discriminate lithium isotopes and to determine if one the lithium isotopes is retired from the dissolution with preference. Additionally, the capacity of *Chamydomonas reinhardtii* to uptake lithium was also explored. Results exhibited two interesting and different behaviors of *C. reinhardtii* respecting to lithium.

### Lithium uptake

**Fig. 1:**
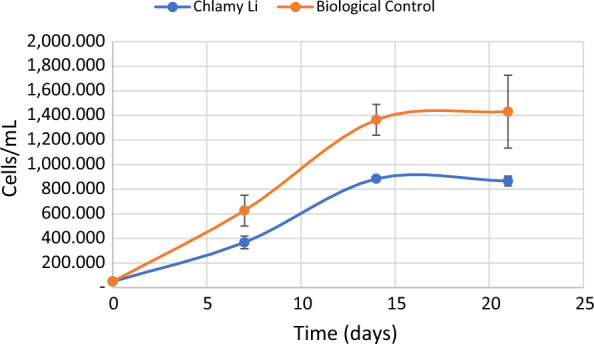
Growth rate of *Chlamydomonas reinhardtii* in presence of 5 mg/L Li (ChlamyLi) and absence of Li (Biological Control)

*Chamydomonas reinhardtii* cultures containing 5 × 10^4^ cell/mL were able to grow in presence of 5 mg/L (0.7 mM) of Li (Table 1). The clone had not been previously exposed to lithium, except for traces in the culture media. Lithium affected the cellular growth (Fig. 1), and certain slowing effect was observed. It is described that the presence of 50 mM of lithium in *Chlamydomonas reinhardtii* cultures blocks flagellar motility [27] and also that 20 mM of LiCl provoke ciliary lengthening [28] which is related to ciliopathy that may affect growth and cell survive conditions. Although the lithium concentration in trials is under the level described in these publications, this fact might be contributing to the slowing down effect observed in *Chamydomonas reinhardtii* growth.

**Table 1:** Growth of *Chlamydomonas reinhardtii* in presence of 5 mg/L of Li (ChlamyLi) and without lithium (Biological Control)

Time (days)	Replicates	ChlamyLi	Biological control
		Number of cells/mL	
0	1	50,000	50,000
	2	50,000	50,000
	3	50,000	50,000
**Mean**		50,000 ± 0,000	50,000 ± 0,000
7	1	310,625 ± 28,382	564,900 ± 37,988
	2	410,725 ± 38,259	770,975 ± 79,873
	3	381,600 ± 40,416	544,150 ± 26,650
**Mean ± SD**		367,650 ± 51,487	626,675 ± 125,397
14	1	884,850 ± 163,490	1,244,525 ± 101,420
	2	865,525 ± 119,714	1,356,475 ± 171,118
	3	905,875 ± 77,589	1,493,900 ± 347,789
**Mean ± SD**		885,417 ± 20,181	1,364,967 ± 124,904
21	1	857,850 ± 31,635	1,473,450 ± 203,178
	2	911,025 ± 225,138	1,116,250 ± 349,597
	3	832,250 ± 62,113	1,704,150 ± 770,737
**Mean ± SD**		867,042 ± 40,184	1,431,283 ± 296,210

In principle, lithium is not a relevant element for living organisms as other as S, P, C, O, H or Mg, Ca, Na and K (between metals). However, there is described the interaction between cells and lithium with consequences on cell function that vary from toxic to beneficious effects, depending upon its concentration or the exposure [29]. Lithium may bind to DNA [30] and additionally the physico-chemical characteristics of this element, next to Na^+^, K^+^, Ca^2+^ or Mg^2+^ let frequently allows its replacement in organisms. This has been observed in *Salmonella typhimurium, Escherichia coli* and *C. elegans* cultures [31]. Lithium may also be incorporated in cellular structures as survival strategy under stress surrounding conditions. Microorganisms use different mechanisms as biosorption of metals to the cell wall (ion exchange, complexation, adsorption, microprecipitation) or bioaccumulation inside the cell through active transport or sequestration to survive in environments with a high presence of metals [32, 33].

**Fig. 2:**
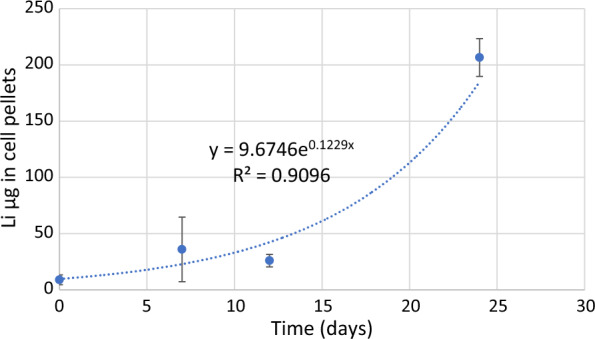
*Chlamydomonas reinhardtii* lithium uptake

Despite the possible toxic effect of lithium, cultures of ChlamyLi containing 5 × 10^4^ cells/mL recovered lithium from solution with a kinetic of 8.23 µg/day. At the starting, just to put in contact cells and Li at the solution, 8.95 µg/L was uptaken. This quantity increased with time up to reach 206.66 µg/L, the final point of the trial. The lithium capture data adjust to an exponential model (*y* = 9.6746. e^0.1229x^, *R*^2^ = 0.9096) (Fig. 2) while the cellular of microalgae arises the stationary phase on day 14th. The rate of lithium capture was 9.41 µg/day. Lithium uptake increased as the concentration of cells, so capture is a process dependent to the quantity of biomass present in the culture. Throughout the 21 days of the experiment it was not reached the maximum capture of Li, then it is possible that this yield could be improved if left longer. Habitual limitations of some biotechnological processed related to microbial uptake of metals such as desorption has not been detected in these trials.

The capacity of *Chlamydomonas reinhardtii* to uptake lithium opens the possibility of treating contaminated environments with microalgae. The biogeochemical cycle of lithium is being altered as a consequence of anthropogenic activity [34]. It is estimated that more than 1,000 × 10^9^ g/year of Li are human mobilized from the Earth's crust. So, a process based on the lithium uptake ability of *Chlamydomonas reinhardtii* might be directed to recover a spoiled element that will be essential from the point of view of future sustainable mobilization (electric cars) and as an alternative energy (nuclear fusion energy).

### Microalga discrimination of lithium isotopes

**Fig. 3:**
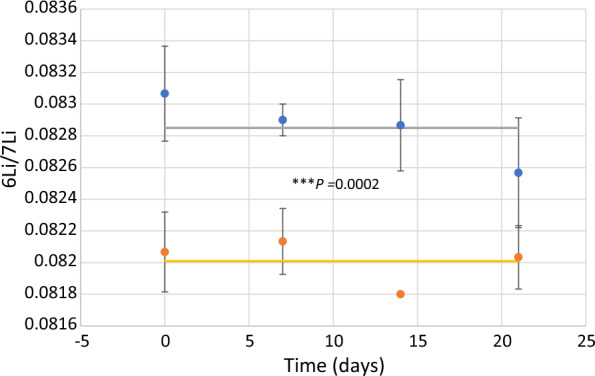
^6^Li/^7^Li in pellets (blue circles) and supernatants (red circles) in *Chamydomonas reinhardtii* cultures. Solid lines correspond to the mean of the ^6^Li/^7^Li in pellets (grey) and supernatants (yellow), respectively. Significant differences of the ^6^Li/.^7^Li value in pellets and supernatants according to t-test (*t* = 5.61, *df* = 11, *p* = 0.0002,  < 0.001)

In relation to isotopic ratio, a very interesting result was achieved: lithium uptake was accomplished with the ^6^Li and ^7^Li discrimination with a microalgal preference for the lighter isotope resulting that *Chlamydomonas reinhardtii* pellets were enriched in ^6^Li; the discrimination factor, δ^6^ value, calculates according to Eq. 3 remained constant around 10.2652 ± 2.9674 throughout the 21 days of the experiment (Table 2). There was a very statistical difference in the ratio ^6^Li/^7^Li between pellets and supernatant, *t-test* (*t* = 5.61, *df* = 11, *p* = 0.0002) (Fig. 3).

**Table 2:** ^6^Li/^7^Li ratio and ^6^δ in *Chlamydomonas reinhardtii* cultures and chemical control experiments

Time (days)	Replicates	Li mass in cells pellet µg	^6^Li/^7^Li ± SD *Chlamydomonas reinhardii*		^6^Li/^7^Li ± SD Chemical control	^6^δ(‰) Pellet/supernatant	^6^δ(‰) Pellet/chemical control
			Pellet	Supernatant			
0	1	8.4	0.0827 ± 0.0011	0.0821 ± 0.0015	0.0821 ± 0.0012	7.3081	7.3081
	2	12	0.0826 ± 0.0008	0.0822 ± 0.0012	0.0821 ± 0.0013	4.8661	6.0901
	3	5.9	0.0839 ± 0.0014	0.0819 ± 0.0010	0.0823 ± 0.0011	24.4200	19.4410
**Mean ± SD**		8.95 ± 4.31	0.0831 ± 0.0001	0.0821 ± 0.0002	0.0822 ± 0.0001	12.1981	10.9533
7	1	26	0.0824 ± 0.0015	0.0823 ± 0.0008	0.0822 ± 0.0017	1.2150	2.4330
	2	68	0.0834 ± 0.0013	0.0820 ± 0.0012	0.0820 ± 0.0011	17.0731	17.0731
	3	13	0.0829 ± 0.0014	0.0821 ± 0.0011	0.0821 ± 0.0015	9.7442	9.7442
**Mean ± SD**		36 ± 28.74	0.0829 ± 0.0005	0.0822 ± 0.0001	0.0821 ± 0.0001	9.3344	9.7442
14	1	20	0.0831 ± 0.0008	0.0819 ± 0.0014	0.0823 ± 0.0010	14.6520	9.7205
	2	27	0.0825 ± 0.0013	0.0815 ± 0.0014	0.0823 ± 0.0013	12.2699	2.4301
	3	31	0.0830 ± 0.0013	0.0820 ± 0.0014	0.0820 ± 0.0007	12.1951	12.1951
**Mean ± SD**		26 ± 5.56	0.0829 ± 0.0003	0.0818 ± 0.0003	0.0822 ± 0.0001	13.0399	8.1103
21	1	203	0.0827 ± 0.0014	0.0820 ± 0.0013	0.0820 ± 0.0013	8.5365	8.5365
	2	225	0.0824 ± 0.0014	0.0819 ± 0.0009	0.0821 ± 0.0009	6.1050	3.6540
	3	192	0.0826 ± 0.0008	0.0822 ± 0.0011	0.0818 ± 0.0011	4.8661	9.7799
**Mean ± SD**		207 ± 16.80	0.0826 ± 0.0003	0.0821 ± 0.0002	0.0820 ± 0.0001	6.5014	7.3200

**Fig. 4:**
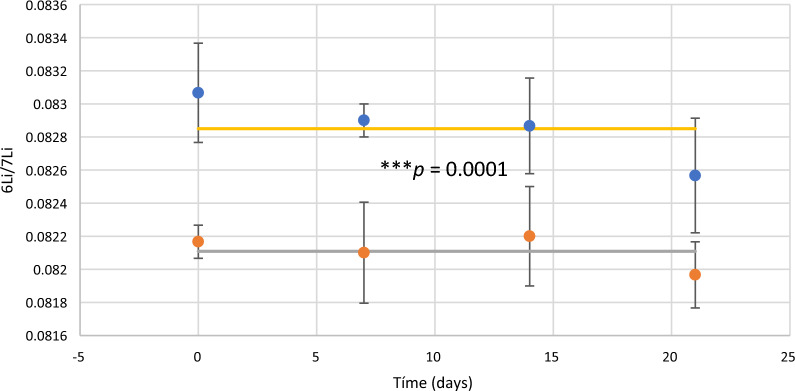
^6^Li/^7^Li in microalgal pellets (blue circles) and chemical control (red circles). Solid lines correspond to the mean of the ^6^Li/.^7^Li in pellets (yellow) and chemical control (grey). Significant differences in the 6Li/7Li in pellets and chemical controls according to *t*-test (*t* = 5.87, *df* = 11, *p* = 0.0001,  < 0.001)

**Fig. 5:**
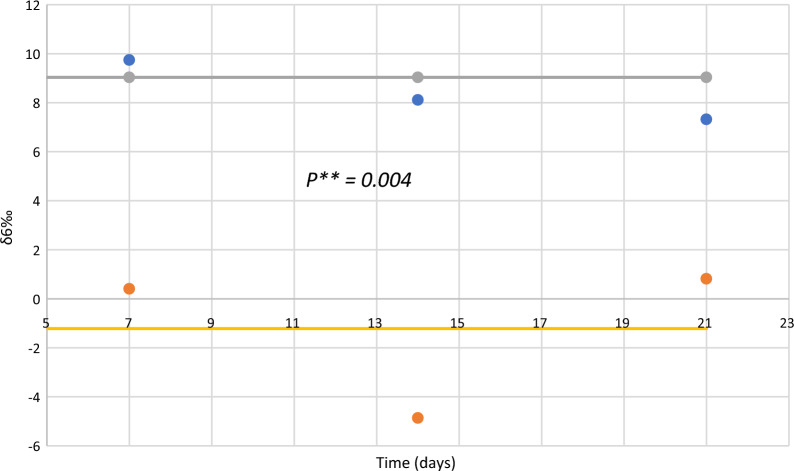
^6^δ value in pellets (blue circles) and supernatant (red circles) in *Chamydomonas reinhardtii* cultures. Promedium pellets (grey) and supernatants (yellow). Significant differences in.^6^δ according to *t*-test (*t* = 4.89, *df* = 4, *p* = 0.004,  < 0.05)

The isotopic relation in cell pellets is also significantly different to the value in chemical control where the relation corresponded to the natural reference maintained stable during the trial, *t-test* (*t* = 5.87, *df* = 11, *p* = 0.0001) (Fig. 4). Thus, ^6^δ estimated in pellets and supernatants confirmed the microalgal enrichment in ^6^Li (Fig. 5).

These results are very promising with the view set on the practical application to the supply of ^6^Li. At the cellular level, ChlamyLi preferentially internalizes ^6^Li, but the affinity or preference for the lighter isotope does not vary over time. It is not necessary to wait 21 days to obtain this value; rather, this fractionation occurs as soon as the microalgae and lithium are put in contact. In contrast with the behavior of *Chamydomonas reinhardtii* related to Li up-taking process, dependent from microalgal growth and then time dependent, isotopic enrichment is a process independent of microalgal growth which would mean that is possible to obtained ^6^Li with a biotechnological processed where time variable would not be determinant because the only limiting factor will be the mass of microalgae and not the growth conditions. For biotechnological applications, microbial biomass may be obtained through a previous step where the best conditions for the growth of microalgae can be provided. Then, biomass will be put in contact with the lithium solution to proceed with isotope separation stage.

The δ^6^ value could be improved by the application of a sequential process in which the enriched ^6^Li/^7^Li in microalgal biomass can be easily recovered (the algal biomass can be degraded under mild calcination or oxidation conditions) and used as starting material in a following cycle, resulting more increase in the ^6^Li/^7^Li ratio.

### Factors affecting microalgal isotopes discrimination

Isotope fractionation mediated by microorganisms can take place through two different mechanisms. One in which fractionation occurs exogenously to the cell and is a consequence of chemical reactions promoted by the organism. An example is the biotic reduction mediated by metal-reducing bacteria which is accomplished with an enrichment in the heavier ^238^U isotope into the solid U(IV) byproduct [35]. In the other alternative mechanism, there is a direct involvement of the cell. This is the case shown by the results obtained with ChlamyLi where the unequivocal participation of microalgae is proved through two evidence: pellets and supernatants have a statistically significative difference on the ^6^Li/^7^Li ratio (Fig. 3); and additionally, there was a statistically difference in the ratio ^6^Li/^7^Li in the chemical control and microalgal pellets (Fig. 4).

To explain why *Chamydomonas reinhardtii* selectively captures ^6^Li it is necessary to understand what differentiates the two isotopes and in what kind of processes this difference may be relevant. As known, isotopes differ in mass number, in the number of neutrons, hence in mass.

Three factors may be influencing the preferential capture of the slighter isotope. The first, kinetic processes are influenced by mass; so, the weak mass difference between ^6^ and ^7^Li (around 16.7%) could explain the greater diffusion rate of ^6^Li and then the preferential uptake for the slighter isotope [36]. Similar result was described by [37] in experiences with cultured human cells and the ^235^U and ^237^U uranium isotopes.

Although our results show that ChlamyLi cells preferentially capture the lighter isotope, this tendency is not general for all biological systems, in fact there are cases in which the organism interlinks the heavier one and even the same organism has different behavior depending on the element. These results have been encountered in bacteria [38] and even in mammal (sheep) cells, where the ^6^Li/^7^Li ratio varied in different tissues [36].

There is a second chemical factor to explain a preferential for the slighter isotope in many biological systems. It is related to the strength of the bond of each isotope in the transition state, i.e., the stability of the transition state. All chemical equilibria (even one as simple as the dissolution of CO_2_ in water) are accompanied by effects on isotopic ratios. When two isotopes (or more) are available for a reaction, each one will result into two different transition states. The bond energy between each isotope and another atom (in the transition state) varies depending on the isotope. The binding energy is higher in the case of heavier isotopes, which slows down their reactivity [39].

In cases where the fractionation is in favor of the heavier element, a mechanism based exclusively on mass-dependent kinetic or on the bond energy in transition state complexes does not explain the results, so other aspects related to the equilibrium between isotopes prior to capture should also be considered. In the complex environment surrounding a cell, a large number of compounds with a potential metal chelating effect may be present, conditioning the availability of an isotope depending on the affinity to bind to a chelating compound. Our case is simple in terms of the quantity and diversity of compounds present at the starting moment. BG-11 is a culture media based on salts (without carbon compounds) so the possibility of differential availability of the two isotopes in the reaction medium (culture medium) does not seem relevant under our conditions.

In another experiments of our group lead to determine the possibilities of uranium fractionation mediated by microalgae [24], a similar behavior as lithium was observed by employing two different microalgae strains: microalga internalized with preference the lighter isotope. So in the case of one the heaviest elements of the Periodic Table such is U, and one of the slighter, Li, the behavior follows the same tendency: microalga uptake the slighter isotope with preference with a delta independent of the growth. So, microalgae can distinguish between isotopes with mass differences as small as 1.3% in the case of uranium and 16.7% in lithium.

Our results demonstrate that the Chlorophyte *Chamydomonas reinhardtii* discriminate the two lithium isotopes and uptake with preference the lighter ^6^Li, through a mass-dependent mechanism with an enrichment of δ^6^ of 10,0299 ± 3,3072 ‰. This result shows a new conceptual approach to get ^6^Li, the fuel indispensable to reach the objectives set out in the ITER project in which it has been estimated that ^6^Li enrichment will be necessary from 7.59% naturally present to at least a 10%. The option presented here, based on microalgal activity has the advantages of all processes based on microbial activity: low energy and material requirements and economic in waste generation. Undoubtedly, the first step to develop a new alternative to obtain the necessary ^6^Li to the fusion processes was achieved.

## Conclusions

Fusion energy is an alternative to those based in the combustion of fossil fuels. To implement fusion energy is necessary ^6^Li isotope. The methods developed up to the moment, based on a physico-chemical approaches do not achieve the desired results. This study reveals that a microalgal culture of *Chlamydomonas reinhardtii* achieved an enrichment in ^6^δ(Li) of 10.029 ± 3.307 through a process independent of the cellular growth. Along this process, the strain recovered up 0.206 mg/L of lithium. All benefits associated to the biotechnological processes accompanied this alternative which has the synergic possibility of removal lithium from polluted environments.

## Data Availability

Data will be available upon request.
